# Development of MEMS Airflow Volumetric Flow Sensing System with Single Piezoelectric Micromachined Ultrasonic Transducer (PMUT) Array

**DOI:** 10.3390/mi13111979

**Published:** 2022-11-15

**Authors:** Xueying Xiu, Haolin Yang, Meilin Ji, Haochen Lv, Songsong Zhang

**Affiliations:** 1 School of Microelectronics, Shanghai University, Shanghai 201800, China; 2 Shanghai Industrial μTechnology Research Institute, Shanghai 201899, China

**Keywords:** PMUT array, ultrasonic flowmeter, cross-correlation

## Abstract

Compared to conventional ultrasonic flowmeters using multiple transducers, this paper reports, for the first time, an airflow volumetric flowmeter using a signal PMUT array to measure the flow rate in a rectangular pipe. The PMUT around 200 kHz is selected to fit the system requirements. All PMUT elements on this single array are then electrically grouped into transmitter and receiver. In order to minimize the crosstalk signal between transmitter and receiver, a phase shift signal is applied at the transmitter to reduce the amplitude of the crosstalk signal by 87.8%, hence, the resultant high sensing resolution. Based on the analog signal extracted from the single PMUT array, a complete flow sensing system is built by using the cross-correlation method and cosine interpolation, whereby the change in flow rate is reflected by the time of flight difference (dTof) recorded at the receiver. Meanwhile, the acoustic path self-calibration is realized by using multiple echoes. Compared with the previously reported MEMS flowmeters with dual or multiple PMUT devices, this paper proposes a single PMUT array flow sensing system, which is able to measure the flow rate changes up to 4 m^3^/h. With the implementation of a single device, the problem of ultrasound device/reflector misalignment during system setup is completely eradicated.

## 1. Introduction

Flow measurement plays an important role in medicine, energy, petrochemical, and other fields [1]. Commonly, flowmeters can be divided into several types such as vortex flowmeters [2], differential flowmeters [3], ultrasonic flowmeters [4], etc. Compared with other flowmeters, ultrasonic flowmeters have the advantages of being non-intrusive, easy operation and installation, and fast response to flow changes [4,5]; therefore, they are widely used in gas and oil pipeline measurement systems. According to the measuring principle, the ultrasonic flowmeter can be divided into transit time ultrasonic flowmeter [5], Doppler ultrasonic flowmeter [6], and cross-correlation flowmeter [7]. Among them, the transition-time ultrasonic flowmeter is the most commonly used because it can easily handle nanosecond intervals, and has high accuracy (calibrated flow rate error < 0.1%) [5,8] and the nonexistence of moving parts [5].

Conventional ultrasonic flowmeters mostly use more than two transducers, one for transmitting and another for receiving echo signals. For the transmitter signal to reach the receiver accurately, the pipe is extended at an angle. Therefore, the devices are often mounted on the pipe in a V, Z, or W shape [9]. However, the turbulent flow and the tendency of accumulating impurities in the extended area of the pipe affect the accuracy of the flowmeter. In addition to this, the use of multiple transducers inevitably introduces installation errors in flow measurement [8]. Therefore, it is a worthwhile direction to remove the pipe extension area and use a single transducer for flow detection. Most of the piezoelectric transducers used in traditional ultrasonic flowmeters are bulk piezoelectric transducers [10]. Due to the limitations of manufacturing technology, the current commercial bulk piezoelectric ultrasonic transducers are relatively large, thus, limiting the size of the pipe. Compared to traditional transducers, micromechanical ultrasonic transducers (MUT) with a smaller volume and lower power consumption are more suitable for small pipe flow detection. In previous reports, Bui et al. [11] and Eovino et al. [12] proposed a method for measuring wind speed using a single MEMS with a reflector. G Bui et al. [11] used two different types of CMUT designs. The device is 10 mm away from the reflector. In pulse-echo mode, the wind speed is reflected by the time of flight (TOF) or the amplitude of the ultrasonic wave at the receiver. However, in order to transmit efficiently, CMUT usually applies a high voltage between the top and bottom electrodes [13,14], therefore increasing the complexity of the circuit. To avoid this problem, Eovino et al. [12] proposed a PMUT single-chip flow sensing system with high sensitivity. The system can measure the airflow direction and the velocity simultaneously. However, the PMUT has a lower bandwidth and a higher quality factor (Q) than the CMUT [15], which results in a long ringing time. PMUT [16] was used by Benjamin E. E to spatially separate the transmitter (Tx) and receiver (Rx) sensor elements to reduce the signal of crosstalk. At a baffle distance of 4 cm, the echo and crosstalk signals can be completely separated. However, in the case of closer distance, the echo signal of the receiver will be hidden in the crosstalk signal, resulting in a large measurement error.

Although the above two methods propose to use a single MUT for wind speed measurement, they only place a baffle on the opposite side of the device as a reflective surface instead of a closed pipe. Using a single transducer in a small pipe is attractive. With the implementation of a single device, the problems of ultrasound device misalignment during system setup are completely eradicated. Meanwhile, removing the pipe extension area where the device is installed can reduce the impact of turbulence on measurement accuracy. Based on the above advantages, this paper first time reports an airflow volumetric flowmeter using a signal piezoelectric micromachined ultrasonic transducers (PMUT) array to measure the flow rate in a small pipe. A single PMUT array is separated from the transmitter and receiver. For the problem of a large crosstalk signal, a phase shift signal is applied at the transmitter to improve the contrast between the echo signal and the crosstalk signal. In addition, the relationship between the flow rate and dTof is discussed, and according to the echo signal of a single PMUT array in the pipe, a complete flow sensing system is built using the cross-correlation method and cosine interpolation. Meanwhile, due to the improvement of contrast between the echo signal and crosstalk signal, the calibration of the acoustic path can be easily achieved by using multiple echo signals in the pipe.

## 2. Measurement Principle

### 2.1. The Method

**Figure 1:**
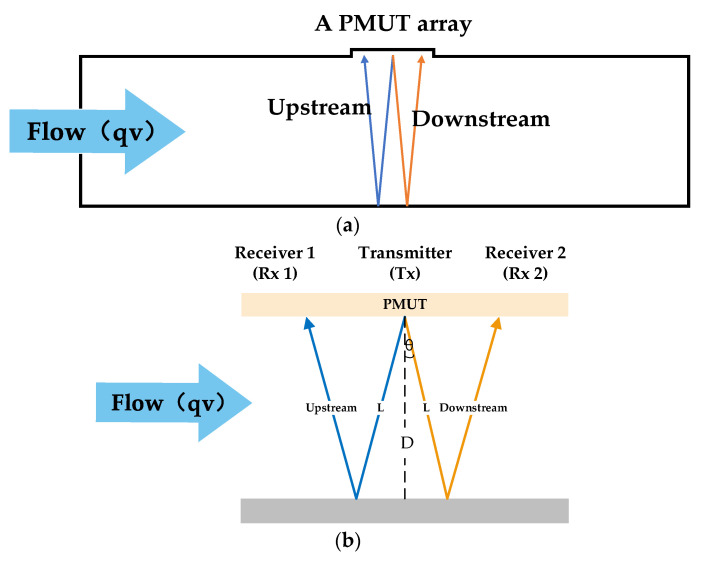
Schematic of the proposed flow sensor. (**a**) Schematic diagram of the flow sensing system pipe. (**b**) Schematic diagram of ultrasound transmitting and receiving in the pipe.

A single PMUT array is symmetrically divided into one transmitter and two receivers. Since the smooth plane of the wall, the received echo signal is formed by reflections from different points [17], as shown in Figure 1. In Figure 1a a single PMUT array is mounted on the pipe, and the center of the array emits ultrasound and the elements on either side of the array receive it. The schematic diagram of ultrasonic wave transmitting and receiving is shown in Figure 1b. Theoretically, the ultrasonic sound path is the same in the zero-flow situation, so there is no dTof between the two receivers. As the flow rate changes, the ultrasound beam is deflected, thus generating a dTof between the receivers. The relationship between dTof(∆*t*) and flow rate can be calculated from Equations (1)–(5).

(1) $$t_{up}=\frac{2L}{c-vsin\theta }$$

(2) $$t_{down}=\frac{2L}{c+vsin\theta }$$

(3) $$\Delta t=t_{up}-t_{down}=\frac{4vLsin\theta }{c^{2}-v^{2}sin^{2}\theta }$$

where *c* is the sound velocity, *L* is the half distance of the acoustic path affected by the fluid flow, *θ* is the angle between the acoustic path and the pipe axis and *v* is the average velocity of fluid flow. Since *v* << *c*, the ∆*t* can be simplified as (4):

(4) $$\Delta t=\frac{4vLsin\theta }{c^{2}}$$

(5) $$v=\frac{c^{2}\Delta t}{4Lsin\theta }$$

The volumetric flow rate is defined by Equation (6):

(6) $$qv=k_{c}Sv=k_{c}S\frac{c^{2}\Delta t}{4Lsin\theta }$$

where *S* is the cross-sectional area of the pipe. *k_c_* is a calibration factor related to the flow state in the pipe. When the Reynolds number is less than 2300, there is laminar flow in the pipe and the calibration factor is 3/4. At Reynolds numbers greater than 2900, the pipe is in turbulent flow, *k_c_* = 2n/(2n + 1). For a smooth pipe, *n* can be expressed by Equation (7) [18], *Re* is the Reynolds number, calculated as (8):

(7) $$n=2log10\left(\frac{Re}{n}\right)-0.8$$

(8) $$Re=\frac{\rho vd}{µ}$$

where *ρ* and *μ* are the density and dynamic viscosity of fluid flow, respectively. *V* is the mean velocity of flow in a pipe. Generally, the *d* of the rectangular channel can be defined as (9) [19]:

(9) $$d=4\frac{A}{L}=2\frac{ab}{a+b}$$

where *A* indicates the cross-sectional area of the rectangular pipe, and *L* is the section circumference of the rectangular pipe. *a* and *b* are the width and length of the section, respectively. In brief, the state of airflow in the pipe can be inferred through the calculation of *Re*, and then the calibration factor can be determined to calculate the flow rate *qv*.

### 2.2. Differential Time of Flight Measurement

The flow measurement is related to the accuracy of the dTof measurement. During the measurement, the transmitter sends out ultrasonic waves. The echo signals of the two receivers are sampled as x[n] and y[n]. Theoretically, there is a high degree of similarity between the echo signals, the main difference is the time deviation generated. Therefore, high accuracy can be achieved using the cross-correlation method. However, the dTof values are usually on the nanosecond and require high hardware specifications. Cosine interpolation is further used to reduce hardware demands. The dTof is calculated using the method in Formulas (10)–(14):

(1) Use the cross-correlation method to calculate Rxy[m] (Equation (10)) and find the index point M for the maximum value.
(2) Find Rxy[M], Rxy[M − 1], Rxy[M + 1], cosine interpolation of the three points to derive the offsets δ [20], (Equations (11)–(13)).
(3) Calculate the final dTof using the offset, M, and sample rate fs (Equation (14)).

(10) $$Rxy\left[m\right]=\overset{\infty }{\underset{-\infty }{\sum }}x\left[n\right]y\left[n-m\right]$$

(11) $$\omega 0=cos^{-1}\left(\frac{Rxy\left[M-1\right]+Rxy\left[M+1\right]}{2Rxy\left[M\right]}\right)$$

(12) $$\theta =tan^{-1}\left(\frac{Rxy\left[M-1\right]-Rxy\left[M+1\right]}{2Rxy\left[M\right]\sin\left(\omega 0\right)}\right)$$

(13) $$\delta =-\frac{\omega 0}{\theta }$$

(14) $$dTof=-\frac{\delta +M}{fs}$$

## 3. The Design of PMUT Array

In gas flowmeters, the acoustic and thermodynamic properties of the gas limit the resonant frequency of the transducer [21]. The ultrasonic attenuation is lower at transducer frequencies below 150 kHz and higher overall at frequencies above 500 kHz [21]. Considering ultrasonic attenuation, this paper uses transducers with a resonance frequency of around 200 kHz.

**Figure 2:**
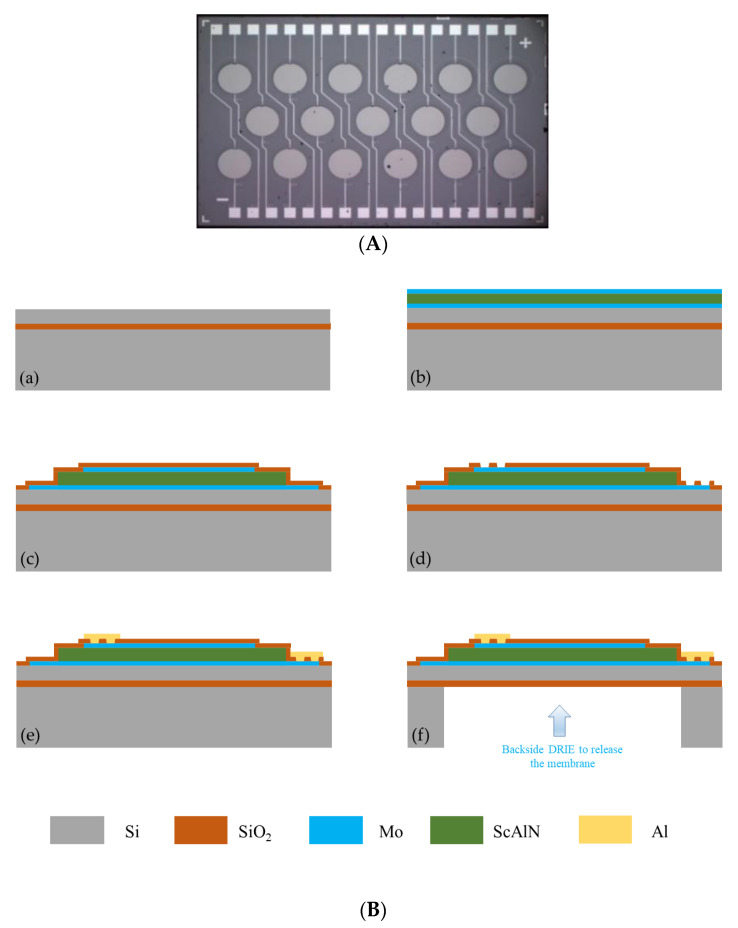
The PMUT array: (**A**) cross-sectional view. (**B**) Process flow.

The PMUT used in this paper is composed of a thin film of a ScAlN piezoelectric layer sandwiched between two molybdenum (Mo) electrodes and a silicon (Si) passive layer. The array includes 17 individual PMUTs, as shown in the light microscope image Figure 2A. Manufactured overall dimensions of 3.1 × 4.7 mm^2^. The corresponding geometric parameters of the PMUT are summarized in Table 1. When an appropriate AC signal is applied to the top and bottom electrodes, the diaphragm will vibrate and transmit ultrasound waves. Conversely, the electrodes will detect an electrical signal when the ultrasonic waves hit the diaphragm.

The process flow of the PMUT array is shown in Figure 2B. (a) A single-sided polished SOI wafer was customized. (b) A multilayer bottom Mo electrode, ScAlN layer, and top Mo electrode were sputtered on the SOI wafer. (c) The bottom Mo electrode, ScAlN layer, and top Mo electrode were patterned by plasma etching, and oxide layers were deposited to form isolation layers. (d) Through holes for the top and bottom electrodes were designed. (e) Aluminum (Al) wires and bond pads were subsequently deposited and patterned. (f) Deep reactive ion etching (DRIE) was performed from the backside of the SOI wafer to release the patterned film. The whole process was performed at the Shanghai Industrial μTechnology Research Institute (SITRI).

**Figure 3:**
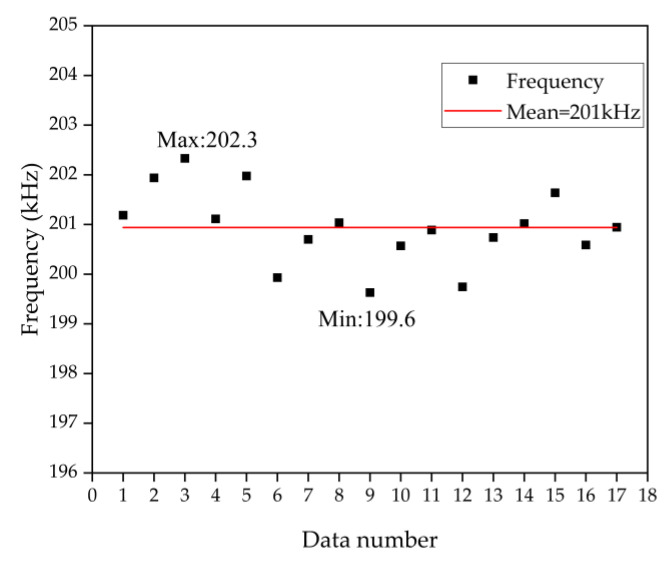
The frequency distribution of the PMUT array, the mean frequency is 201 kHz with a deviation of 1.5 kHz (the standard deviation is 0.732).

**Figure 4:**
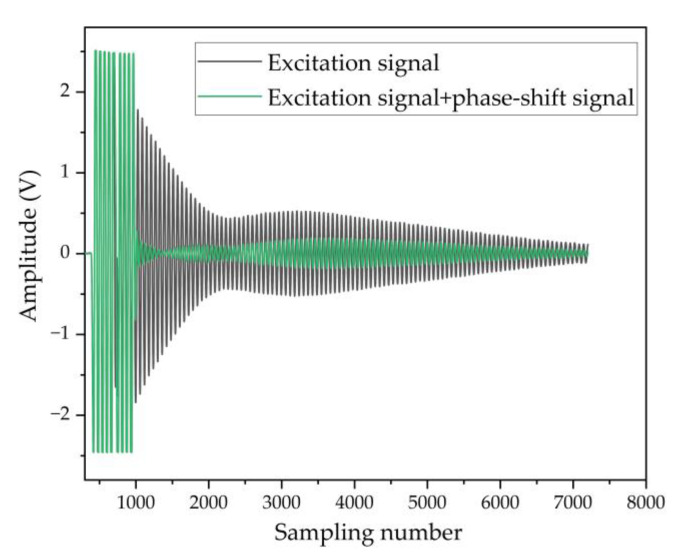
Crosstalk signal at the receiver (black line: there is no phase shift signal at the transmitter; green line: phase shift signal is applied at the transmitter).

The resonant frequency is measured using an impedance analyzer, as shown in Figure 3. The resonant frequency of the array is 201 kHz within a deviation of 1.5 kHz, which is smaller than the bandwidth of approximately 3.19 kHz. As a result, the estimated Q-factor is around 63 and it introduces a relatively long mechanical ringing on the transmitting PMUT elements even after removing the driving signal. When a PMUT array is divided into transmitter and receiver, such ringing-induced crosstalk across the array seriously affects the SNR of the echo signal recorded on the receiver, especially when the echo overlaps with the ringing coupled from the transmitter in case of a shorter acoustic path [12]. As shown in Figure 4, the black line is the crosstalk signal that exists at the receiver when the excitation signal is transmitted. When the reflector is close to the transmitter, the echo signal is hidden in the crosstalk signal, resulting in an acoustic dead zone. By adding a phase shift signal [22,23] at the end of the excitation signal, the crosstalk signal (green line) at the receiver is reduced by 87.8%, improving the contrast between the actual echo signal and the crosstalk noise, so a better receiving SNR is achieved.

**Figure 5:**
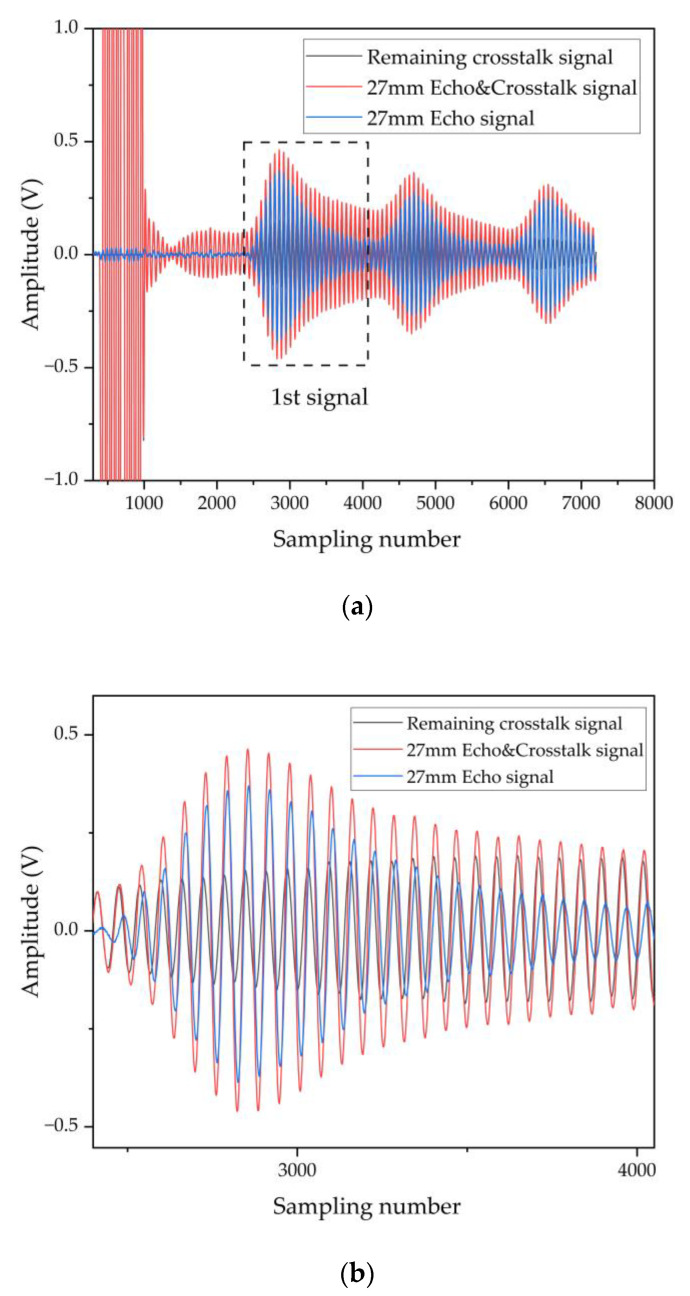
(**a**) Pulse echo measurement at 27 mm in distance and remaining crosstalk signal. (**b**) The detail of 1st signal.

To further determine the position of the signal in the pipe, a baffle is positioned at 27 mm from the transducer. The signal at the receiver is shown as the red line in Figure 5a, which shows a superposition of the echo and crosstalk signals. The crosstalk signal (black line) at the receiver without the baffle is used as a reference. After that, the reference signal is subtracted from the signal at the receiver with the baffle. Finally, the true echo signal is obtained, as shown in the blue line in Figure 5a. The remaining crosstalk signal (black line) causes the echo signal (blue line) to deviate in amplitude and phase, as in Figure 5b. However, the crosstalk signal is determined by the device itself [24,25] and is less affected by changes in flow rate. Therefore, the effect of remaining crosstalk signals does not affect the sensing performance. The variation of the overall signal (Figure 5 (red line)) with flow rate is considered to be the variation of the echo signal (Figure 5 (blue line)) with flow rate.

## 4. Experiment and Discussion

### 4.1. Experimental Setup

**Figure 6:**
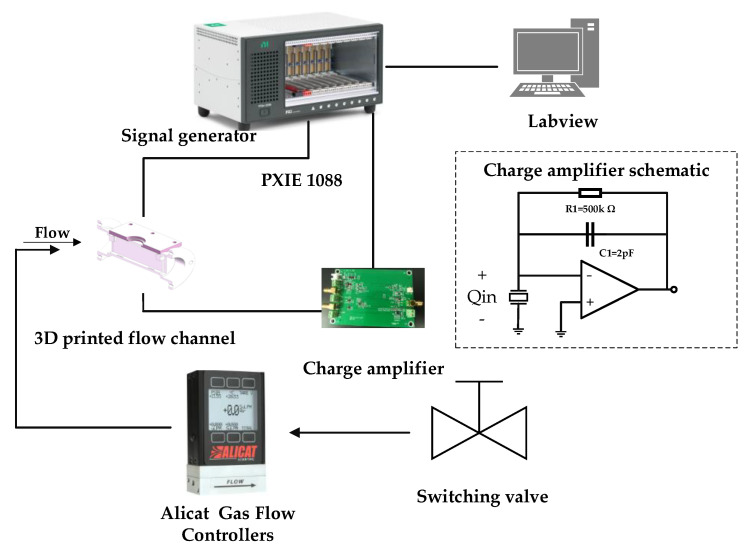
Block diagram of the ultrasonic flowmeter system.

**Figure 7:**
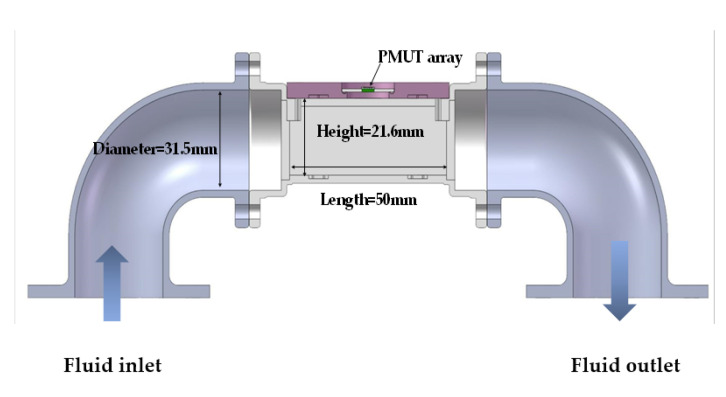
Scheme of the flow channel, the overall dimensions are 50 mm × 8.9 mm × 21.6 mm.

**Figure 8:**
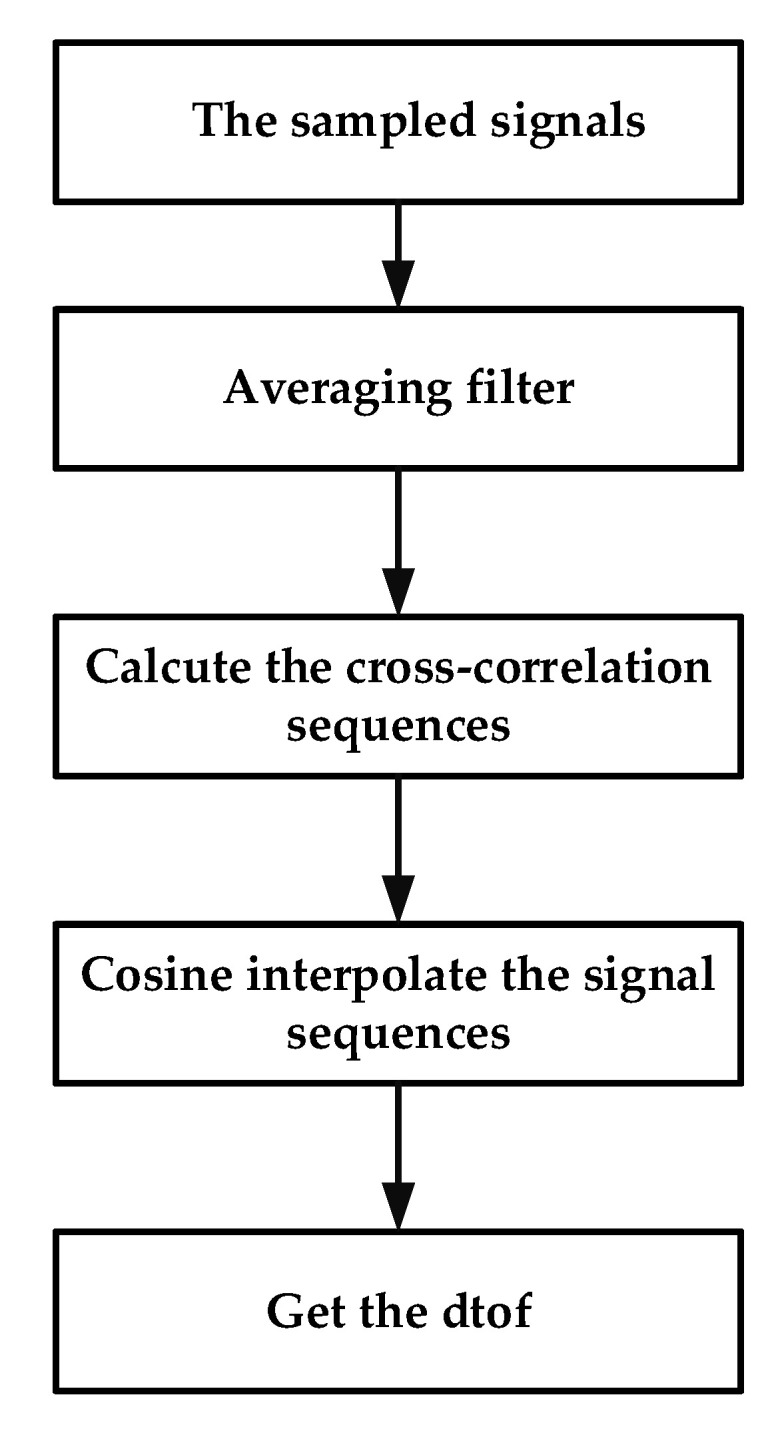
The overall algorithm of the flow sensing system.

The setup for the flowmeter based on PMUT arrays is illustrated in Figure 6. The dimensions of the rectangular flow channel used in this paper (Figure 7) are 50 mm × 8.9 mm × 21.6 mm. Three 0.3 mm wide baffles are located inside the pipe. It can stabilize the flow rate in a pipe and improve measurement accuracy [18]. In addition, a 0.5 mm thick rubber gasket is added between the device and the pipe to ensure a tight seal. PMUT arrays are mounted on PCB by silica gel and then fixed on side of the designed flow channel. A commercial flowmeter (ALICAT, M040-LK2) is used for reference. The valve is turned on to allow the airflow to pass through and ALICAT controls and displays the flow of air. The NI PXIe is used to excite the transmitter with the square wave signal of 10 Vpp at 201 kHz and handle the received signal after charge amplification, and then the time difference between the downwind and upwind TOF are calculated by cross-correlation method and displayed on the UI by a Labview program. The overall algorithm used for the flow sensing system is shown in Figure 8.

### 4.2. Auto-Calibration Technique

**Figure 9:**
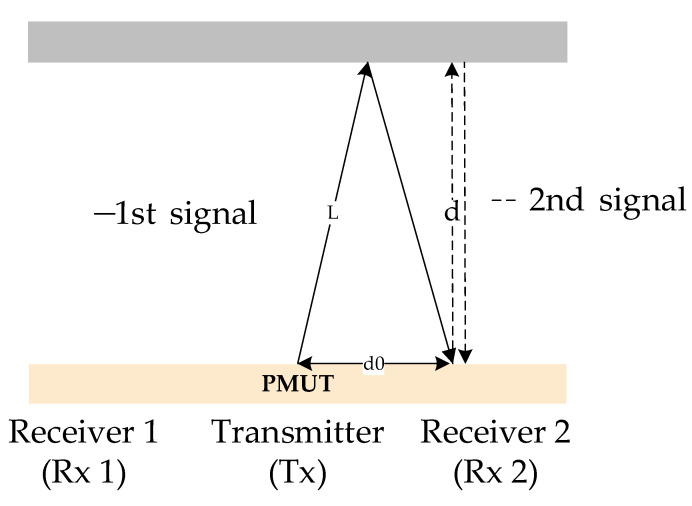
Schematic of the proposed ultrasonic path calibration.

**Figure 10:**
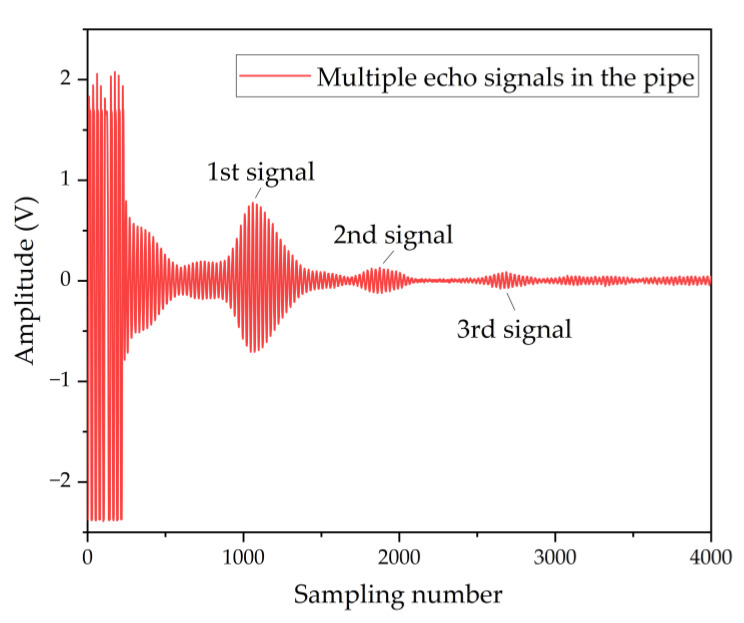
Multiple echo signals in the pipe.

The ultrasonic path *L* is the parameter that is not measured and is involved in the flowmeter. As shown in Figure 9, the centers from the transmitter to the receiver are at a distance *d*0 of 1500 µm. The distance *D* from the device to the tube wall is designed to be 27 mm, so *D* >> *d*0/2. From Equation (15), it can be considered that *D*~*L*. However, the *D* values will be deviated from the design value due to errors in transducer installation. The tof between the excitation signal and the echo signal cannot be measured accurately due to the delay in the system. In contrast, the high similarity between multiple echoes allows a more accurate tof obtained from the cross-correlation method. Multiple echo signals in the pipe are shown in Figure 10. The temperature in the pipe is 23 °C. The tof calculated using the primary and secondary echo signals is 152.622 us. The *D* derived from Equation (16) is 26.4 mm, which is also the ultrasonic path *L*.

(15) $$L=\sqrt{D^{2}+{\left(\frac{d0}{2}\right)}^{2}}$$

(16) $$D=\frac{1}{2}\times c\times tof$$

### 4.3. Flow Measurement

**Figure 11:**
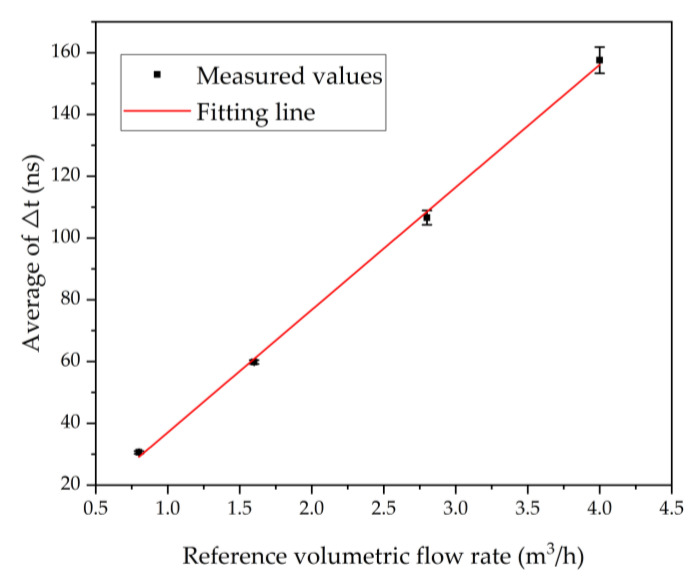
The measured dTof for different volumetric flow rates and the fitting line show the linear relationship.

Before the start of the experiment, it is necessary to determine the standard flow rates. The detection flow rates are 0.8 m^3^/h, 1.6 m^3^/h, 2.8 m^3^/h, 4 m^3^/h, and each measurement lasted 60 s. Figure 11 shows the dTof corresponding to different volumetric flow rates. As predicted by Equation (6), the average of dTof varies linearly with the volumetric flow rates. However, as the flow rate increases from 0.8 to 4 m^3^/h, the variance of dTof varies from 0.433 to 4.247.

**Figure 12:**
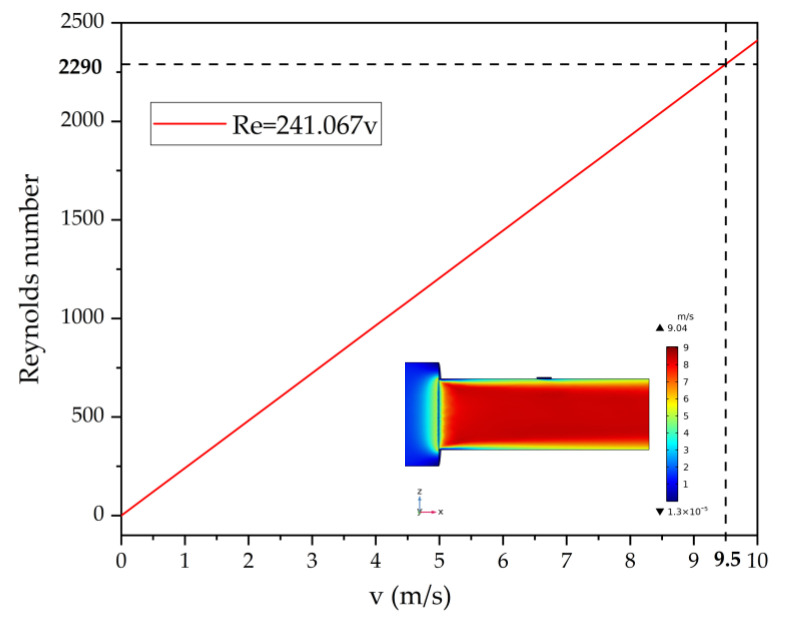
Reynolds number versus flow velocity (CFD simulation is inserted in the figure to represent the flow velocity distribution in the pipe when the flow rate is 4 m^3^/h).

**Figure 13:**
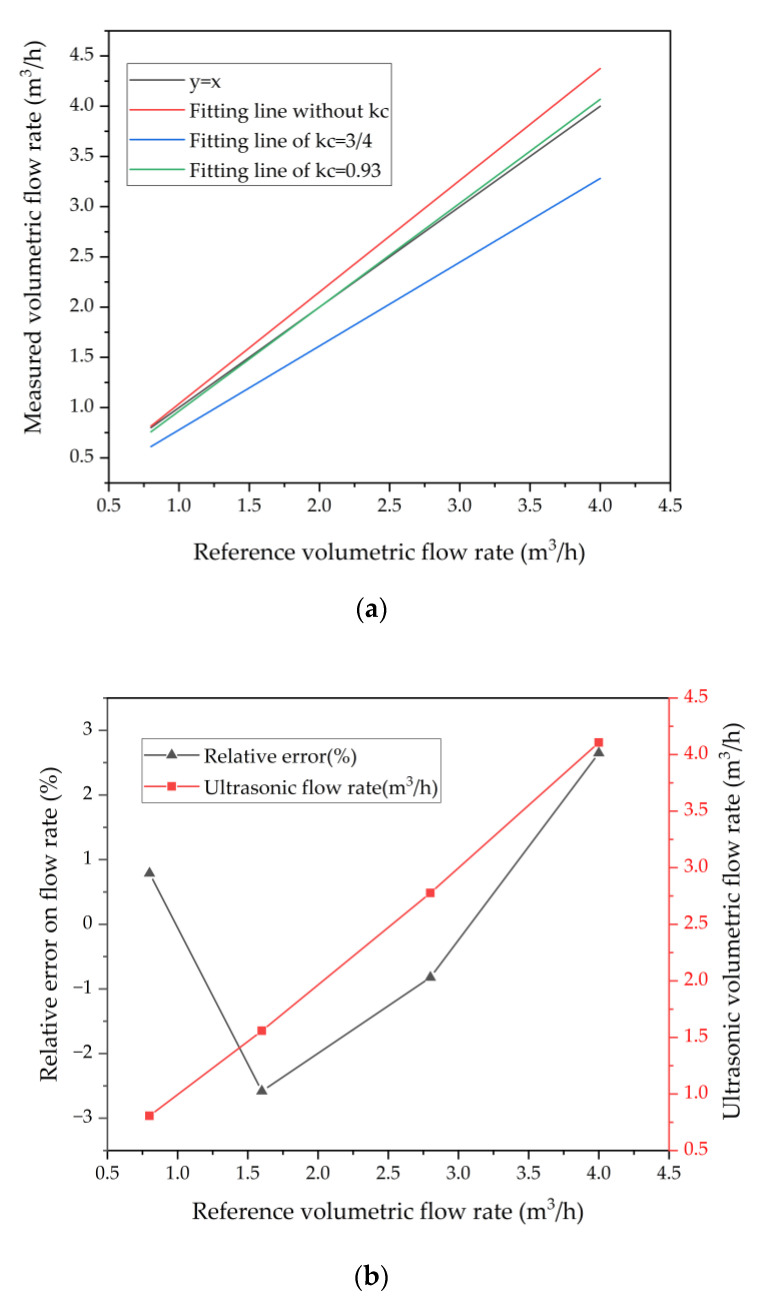
(**a**) The measured volumetric flow rates and the linear fitting results. (**b**) Relative errors on the correctional ultrasonic volumetric flow versus the reference flow rates.

The condition of the fluid in the pipe can be reflected by the Reynolds number. Considering that the pipe is filled with air, ρ and μ in Equation (8) are 1.83 × 10^−5^ Pa·s and 1.205 kg/m^3^, respectively [18]. Due to three baffles dividing the pipe into four 2 mm width channels. The Reynolds number is calculated based on a 2 mm width flow channel. Here a is 2 mm, b is 21.6 mm. The calculated Reynolds number versus flow velocity is plotted in Figure 12. The fluid in the pipe remains laminar at a flow velocity of less than 9.5 m/s. The fluid enters the rectangular pipe from the circular inlet. At a flow rate of 4 m^3^/h, CFD simulations show a maximum velocity of 9.04 m/s at the interface between the circular inlet and the rectangular pipe, which is still below 9.5 m/s. (as shown in Figure 12). The result shows the fluid in the pipe is laminar flow. Theoretically, there will be a fixed calibration factor *k_c_* = 3/4. However, the actual calibration factor deviates from the theoretical value due to errors in device design and installation, pipe manufacture, etc. [10]. This paper uses the method referring to some previous work [9] where the ratio of the reference flowmeter to the ultrasonic flowmeter is considered to be the actual calibration factor. The flow rate Q1 is measured by the ultrasonic flowmeter, while Q2 is measured by the Alicat. The calibration factor is the flow ratio Q2:Q1. From Table 2 it can be seen that the calibration factors are in the range of 0.906 to 0.955 with an average value of 0.93. The ultrasonic flowmeter (Q1) is calibrated using the experimental calibration factor *k_c_* = 0.93. Furthermore, as the flow rate changes, the temperature and pressure in the pipe changes, and in turn affects the speed of sound. The variation of the speed of sound in the pipe is disregarded because of the relatively slight variation of temperature and pressure at flow rates from 0 to 4 m^3^/h. Figure 13a shows the calibrated flow and reference flow. Where the red line is the flow rate after calibration by *k_c_* = 3/4 and there is a significant deviation from the reference value. This phenomenon can be explained by the existence of manufacturing errors [10]. The value corrected using *k_c_* = 0.93 in this paper (blue line) is almost identical to the reference value (black line) As shown in Figure 13b, the relative error of the corrected volumetric flow rate to the reference volumetric flow rate is within 3%.

**Figure 14:**
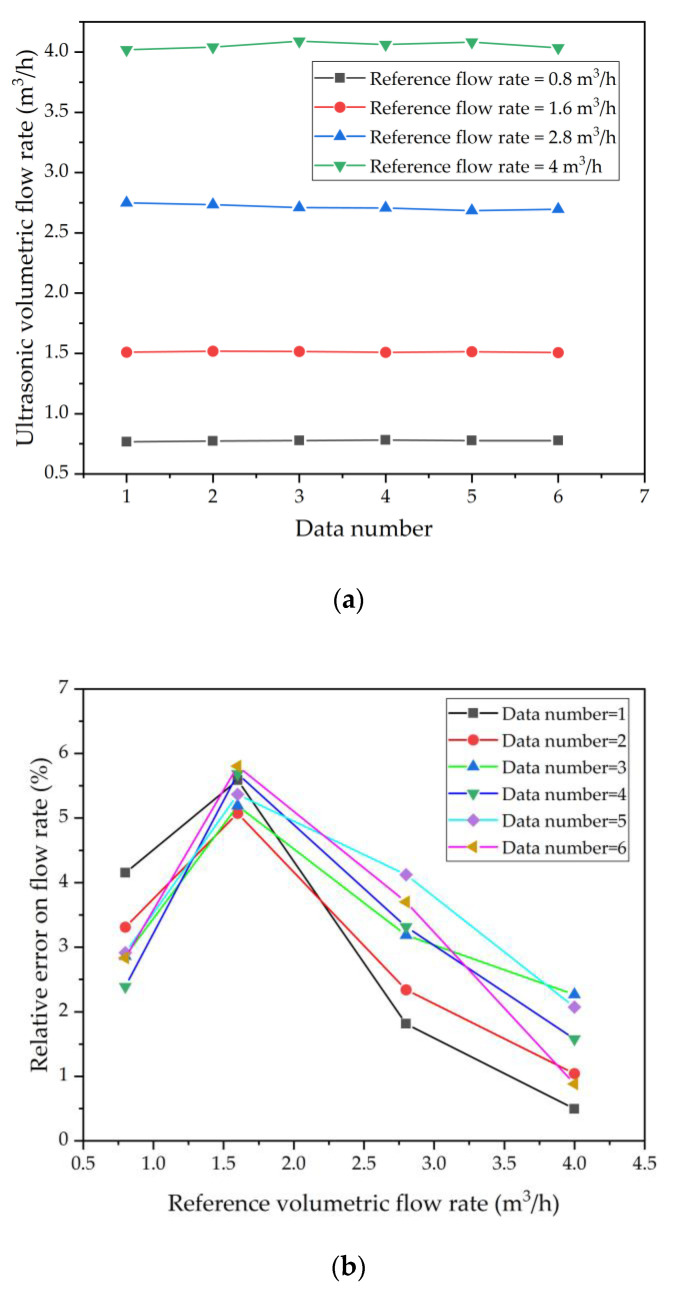

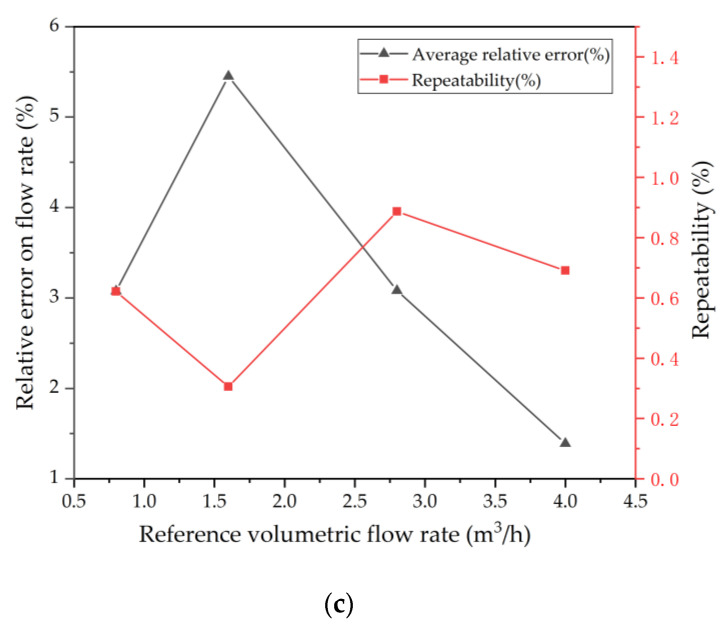
(**a**) The ultrasonic volumetric flow rate after calibration using *k_c_* = 0.93. (**b**) Relative errors on the ultrasonic volumetric flow versus the reference flow rates. (**c**) Average relative error of six measurements and repeatability error.

To determine the relative and repeatability [26] errors of the flow. Six measurements are conducted at one single flow rate and each measurement lasts 60 s. Figure 14a shows the ultrasonic volumetric flow rate after calibration using *k_c_* = 0.93. It can be seen that the error decreases at higher flow rates in Figure 14b. However, since the kc is selected with a mean value, a large error can occur at some flow points (such as 1.6 m^3^/h). The average values of the relative error of the six measurements are shown in Figure 14c and are within 5% relative error, and the repeatability error is less than 0.88%.

This paper investigates the feasibility of using a single PMUT array to measure flow in a pipe. Table 3 gives comparisons with previous work The previously reported wind speed measurement system [11,12] based on a single MUT is not placed in a closed pipe, rather a baffle is placed on the opposite side of the transducer to be used as a reflective plane. CMUT-based anemometers typically require a high DC bias between the top and bottom electrodes, increasing the complexity of the circuit. On the other hand, PMUT flow sensing systems introduce a large measurement dead zone, restricting applications in small pipes. In this paper, a 200 kHz PMUT device is chosen in consideration of the attenuation of ultrasound in the pipe. A single PMUT array is mounted in a modified rectangular pipe, reducing 87.8% of the crosstalk at the receiver using a phase shift signal, and enhancing the contrast between the signal and the remaining crosstalk. Compared to previous applications using two sensors in a circular pipe, this paper achieves a flow measurement of 4 m^3^/h.

## 5. Conclusions

In summary, high volumetric flow rate sensing in a rectangular pipe is accomplished using a single PMUT array with a frequency of about 200 kHz and an overall size of 3.1 × 4.7 mm^2^. The PMUT elements on a single array are grouped into the transmitter and the two receivers. To solve the acoustic dead zone due to the crosstalk coupled between transmitter and receivers, a phase shift signal is applied and a high contrast echo signal is recorded on the receiving terminal for a better system SNR. In addition, the relationship between flow rate and dTof is discussed for a single PMUT array in the pipe. According to the similarity of echo signals, a complete flow sensing system has been developed using a highly noise-resistant cross-correlation method combined with cosine interpolation. At the same time, the acoustic path is calibrated according to the multiple echo signals in the pipe. Compared to previously reported PMUT flowmeters, we successfully demonstrate a volumetric airflow detection up to 4 m^3^/h (~9m/s) in a small pipe using a single PMUT array. A brief summary of the comparison between our works and the literature is tabulated in Table 3. With the significance of a single flow sensing device setup, the common practical problems related to ultrasound device/reflector misalignment due to setup imperfection are completely eradicated.

## Figures and Tables

**Table 1:** Geometric parameters of the PMUT array.

Material	Top Mo	ScAlN	Bottom Mo	Si	SiO_2_	Cavity
Radius (µm)	234					300
Thickness	0.15	1	0.2	4	1	400

**Table 2:** Results of flow rate measured.

No.	Reference Flowmeter (m^3^/h)_Q2	Ultrasonic Flowmeter (m^3^/h)_Q1	Q2:Q1	Q1 × 0.93	Relative Error
1	0.8	0.867	0.92272203	0.80631	0.78875
2	1.6	1.676	0.954653938	1.55868	−2.5825
3	2.8	2.986	0.93770931	2.77698	−0.822142857
4	4	4.415	0.906002265	4.10595	2.64875

**Table 3:** Comparison of reported MUT for airflow applications.

	This Work	Ref. [10]	Ref. [12]	Ref. [11]
Frequency	201 kHz	980 kHz	730 kHz	200 kHz
Transducers	A PMUT array	Two PMUT arrays	A PMUT array	A CMUT array
Pipe’s shape	Rectangular	Circular	Only baffle	Only baffle
Pipe’s height	21.6 mm	10 mm	4 cm	10 mm
Flow rate/Velocity	4 m^3^/h (~9 m/s)	0.56 m^3^/h	5 m/s	10 m/s

## Data Availability

Data are available from the authors on request.
